# Expression of Prostate-Specific Membrane Antigen (PSMA) on Biopsies Is an Independent Risk Stratifier of Prostate Cancer Patients at Time of Initial Diagnosis

**DOI:** 10.3389/fonc.2018.00623

**Published:** 2018-12-20

**Authors:** Marie Christine Hupe, Christian Philippi, Doris Roth, Christiane Kümpers, Julika Ribbat-Idel, Finn Becker, Vincent Joerg, Stefan Duensing, Verena Helena Lubczyk, Jutta Kirfel, Verena Sailer, Rainer Kuefer, Axel Stuart Merseburger, Sven Perner, Anne Offermann

**Affiliations:** ^1^Department of Urology, University Hospital Schleswig-Holstein, Luebeck, Germany; ^2^Pathology of the University Hospital Schleswig-Holstein and Research Center Borstel, Leibniz Lung Center, Luebeck, Germany; ^3^Molecular Urooncology, Department of Urology, University of Heidelberg School of Medicine, Heidelberg, Germany; ^4^Department of Pathology, Klinik am Eichert Alb Fils Kliniken, Goeppingen, Germany; ^5^Department of Urology, Klinik am Eichert Alb Fils Kliniken, Goeppingen, Germany

**Keywords:** disease recurrence, immunohistochemistry, prognostic biomarker, prostate biopsy, prostate cancer, prostate-specific membrane antigen

## Abstract

**Background:** Stratifying prostate cancer (PCa) patients into risk groups at time of initial diagnosis enabling a risk-adapted disease management is still a major clinical challenge. Existing studies evaluating the prognostic potential of PSMA (prostate-specific membrane antigen) for PCa were performed on radical prostatectomy specimens (RPE), i.e., decision making for disease management was already completed at time of sample analysis. Aim of our study was to assess the prognostic value of PSMA expression for PCa patients on biopsies at time of initial diagnosis.

**Methods:** PSMA expression was assessed by immunohistochemistry on 294 prostate biopsies with corresponding RPE, 621 primary tumor foci from 242 RPE, 43 locally advanced or recurrent tumors, 34 lymph node metastases, 78 distant metastases and 52 benign prostatic samples. PSMA expression was correlated with clinico-pathologic features. Primary endpoint was recurrence free survival. Other clinicopathologic features included WHO/ISUP grade groups, PSA serum level, TNM-stage, and R-status. Chi-square test, ANOVA-analyses, Cox-regression, and log-rank tests were performed for statistical analyses.

**Results:** High PSMA expression on both biopsy and RPE significantly associates with a higher risk of disease recurrence following curative surgery. The 5-year-recurrence free survival rates were 88.2, 74.2, 67.7 and 26.8% for patients exhibiting no, low, medium, or high PSMA expression on biopsy, respectively. High PSMA expression on biopsy was significant in multivariate analysis predicting a 4-fold increased risk of disease recurrence independently from established prognostic markers. PSMA significantly increases during PCa progression.

**Conclusion:** PSMA is an independent prognostic marker on biopsies at time of initial diagnosis and can predict disease recurrence following curative therapy for PCa. Our study proposes the application of the routinely used IHC marker PSMA for outcome prediction and decision making in risk-adapted PCa management on biopsies at time of initial diagnosis.

## Introduction

Prostate cancer (PCa) is still the most common cancer type among men with more than 200 new diagnoses per 100,000 men/year in Northern and Western Europe (1). The combination of the serum marker prostate specific antigen (PSA), Gleason score of the prostate biopsy, and clinical stage is currently used to stratify patients into different risk groups for biochemical recurrence (1). Recently, a new Gleason Grading System has been introduced by the International Society of Urological Pathology (ISUP) aiming to increase accuracy and thus the prognostic value (2). However, while the new grading system seems to reduce the rate of upgrading from biopsy to corresponding radical prostatectomy specimens (RPE) it does not seem to significantly improve the prognostic value (3). Thus, further diagnostic tools are urgently needed to optimize risk stratification of PCa patients at time of initial diagnosis.

Commonly used diagnostic markers to confirm PCa include PSA, androgen receptor, or prostate-specific membrane antigen (PSMA) (4). PSMA was first described in 1987 and subsequently characterized as a transmembranous glycoprotein exhibiting hydrolytic activity (5, 6). PSMA is expressed in normal, benign and malignant prostate tissues including intraepithelial neoplasia and metastatic specimens (7, 8). Notably, PSMA expression is significantly higher in primary PCa as compared to benign tissue and also significantly higher in lymph node and distant metastases as compared to primary tumors (4).

PSMA already has a variety of clinical implications in the management of PCa (9). In case of biochemical recurrence following curative therapy, a 68Ga-PSMA PET/CT can be performed to detect local and distant cancer sites. PSMA acts as the target for the radiotracer (10, 11). Similarly, in the course of salvage surgery for recurrent PCa it has been demonstrated that intravenous 99mTc-PSMA can intraoperatively increase detection of metastatic lesions using a gammaprobe known as radio-guided surgery (12). PSMA can also be targeted by PSMA ligands for a radioligand therapy, such as Lutetium-177. This therapy is provided in compassionate use programs or applied as last line treatment option for metastatic castration resistant PCa (13–15).

In RPE specimens, PSMA overexpression has already been linked to an unfavorable biochemical recurrence free survival rate (16–18). In prostate biopsy and RPE specimen, PSMA expression also significantly correlates with Gleason Score (18) implying a potential prognostic value for PSMA in prostate biopsy as well.

Notably, above mentioned studies evaluating the prognostic potential of PSMA for PCa outcome performed analyses at time of therapy by using RPE specimens, i.e., the assessed PSMA level has no impact on treatment decision.

The aim of the present study however was to assess the independent prognostic value of PSMA expression on prostate biopsy specimens enabling a future PSMA based risk stratification of PCa patients with localized disease at time of initial diagnosis.

## Materials and Methods

### Cohort Description and Material

The cohort used in this study includes 294 preoperative biopsies (Biopsy Cohort), 621 primary tumor foci from 242 patients (RPE Cohort), 43 locally advanced or recurrent tumors obtained from transurethral resection of the prostate, 34 lymph node metastases, and 52 benign prostatic samples from patients who underwent surgery for prostate cancer in the Hospital of Goeppingen, Germany between 2002 and 2014. From 124 patients, corresponding pre-operative needle biopsy and RPE specimen were available. Survival analysis considering biopsies and RPE specimens was performed by including all patients with available follow-up data. Additional 78 distant metastases of patients who were treated at the University Hospital Schleswig-Holstein, Campus Luebeck, Germany, were included in the present study. Patients' characteristics are presented in Table 1.

**Table 1:** Patient characteristics.

**Parameter**	**Biopsy cohort (*n* = 246)**	**Radical prostatectomy cohort (*n* = 242)**
**WHO grade group [*****n*** **(%)]**		
1	104 (42.3)	93 (38.4)
2	45 (18.3)	89 (36.8)
3	21 (8.5)	36 (14.9)
4	30 (12.2)	12 (5.0)
5	17 (6.9)	12 (5.0)
Unknown	29 (11.8)	0
**T [*****n*** **(%)]**		
2	–	163 (67.4)
3a	–	30 (12.4)
3b	–	29 (12.0)
4	–	2 (0.8)
Unknown		18 (7.4)
**N [*****n*** **(%)]**		
0	–	212 (87.6)
1	–	24 (9.9)
Unknown		6 (2.5)
**M [*****n*** **(%)] synchronous**		
0	–	237 (97.9)
1	–	0
Unknown		5 (2.1)
**R [*****n*** **(%)]**		
0	–	173 (71.5)
1	–	56 (23.1)
Unknown		13 (5.4)
Initial PSA [mean ± SD; ng/ml]	9.54 ± 8.28	22.86 ± 207.60
Disease recurrence^*^ **[*****n*** **(%)]**		
0	167 (67.9)	180 (74.4)
1	79 (32.1)	62 (25.6)
Unknown	0	0
**Death (overall survival) [*****n*** **(%)]**		
0	232 (94.3)	226 (93.4)
1	9 (3.7)	11 (4.5)
Unknown	5 (2.0)	5 (2.1)
**PSMA mean [*****n*** **(%)]**		
0 (negative)	32 (13.0)	33 (13.6)
1 (low)	103 (41.9)	126 (52.1)
2 (medium)	83 (33.7)	67 (27.7)
3 (high)	28 (11.4)	16 (6.6)
Unknown	0	0
**PSMA max [*****n*** **(%)]**		
0 (negative)	–	33 (13.6)
1 (low)	–	90 (37.2)
2 (medium)	–	94 (38.8)
3 (high)	–	25 (10.3)
Unknown		0
Follow-up [median; min; max; months]	72.7; 0.7; 152.6	88.4; 0.6; 169.1

* *Defined as PSA relapse following radical prostatectomy*.

Disease recurrence was defined as biochemical recurrence (PSA increase above the post-operative nadir following RPE) and used as endpoint for survival analysis.

Ethical approval for the present study was obtained from the Internal Review Board of the University Hospital Schleswig-Holstein, Campus Luebeck.

Immunohistochemical (IHC) analysis of tissues except biopsies was performed on tissue microarrays (TMA). Briefly, formalin-fixed paraffin-embedded tissues were cut in 4-μm thick sections, mounted on slides and relevant tissue regions were circled by a pathologist. Three representative cores of the circled regions measuring 0.6 mm in diameter from each sample were assembled into tissue microarray blocks using a semiautomatic tissue arrayer. For more details see (19).

### Immunohistochemistry

IHC staining was performed using the Ventana Discovery automated staining system (Ventana Medical System). In brief, slides were incubated at room temperature with primary antibody: anti-PSMA rabbit monoclonal antibody (SP29), SPING Bioscience, and detected with the ultraView Universal DAB Detection Kit (Ventana Medical System).

**Figure 1:**
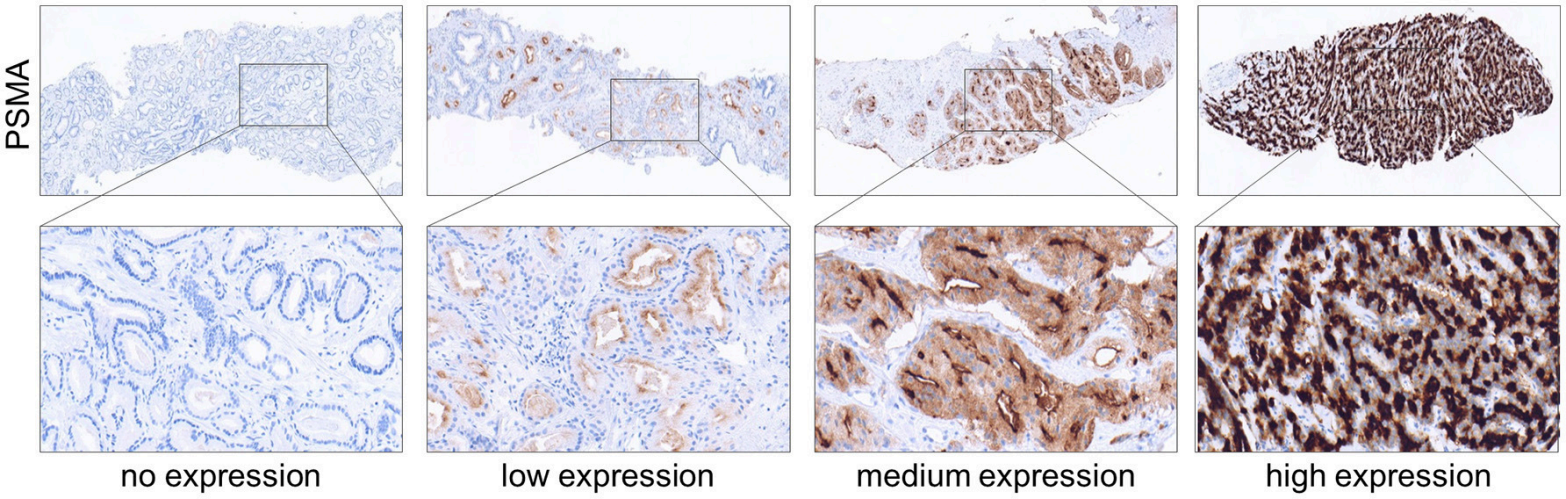
Immunohistochemical images showing PSMA negative, low, medium and high expressing prostate tumors obtained from needle biopsies. **(Upper)** Magnification 4x. **(Lower)** Magnification 40x.

Membranous PSMA expression was categorized according to its intensity and scored as the following: 0 (no expression), 1 (low expression), 2 (medium expression), and 3 (high expression). Images showing examples for different PSMA scores are shown in Figure 1. IHC analysis was performed by two independent pathologists. In cases of discrepancy, the two pathologists agreed to a consensus.

For each patient, only the needle biopsy with highest grade group was selected for IHC staining. In cases with consistent grade group between biopsies, the core with highest tumor volume was selected for IHC. In order to take PCa multifocality into account, within RPE specimens, up to 5 largest tumor foci were represented on tissue microarrays. For each patient, the mean PSMA score between all tumor foci, as well as the maximum PSMA score out of the assessed tumor foci were used for further statistical analyses.

Out of 294 biopsies that have been stained, 276 (94%) were suitable for analysis. Following cutting for IHC 29 (10%) biopsies lacked tumor tissue.

### Statistics

Cox proportional-hazards regression models were performed to investigate the association between PSMA expression and iPSA. For multivariate analysis, PSMA expression was adjusted to grade group on biopsy and initial PSA (iPSA) for analysis of biopsies, and to T-stage, iPSA, R-status, N-status, and grade group for analysis of RPE specimens. PSMA was defined as a nominal categorical variable. For all multivariable analyses, we defined “PSMA negative” as the reference group.

Kaplan–Meier curves were used to illustrate biochemical recurrence free survival and statistically proved by log-rank test. Chi-Square test was performed to investigate the association between PSMA expression and grade groups, T-stage, N-status, and iPSA, as well as between PSMA on biopsies and RPE specimens. ANOVA was performed to analyze differences in PSMA expression levels in different tissue types.

All statistical analyses were performed using SPSS 20.0. P levels < 0.05 were considered significant.

## Results

### PSMA Expression Increases During PCa Progression and Correlates With Established Prognostic Biomarkers

**Figure 2:**
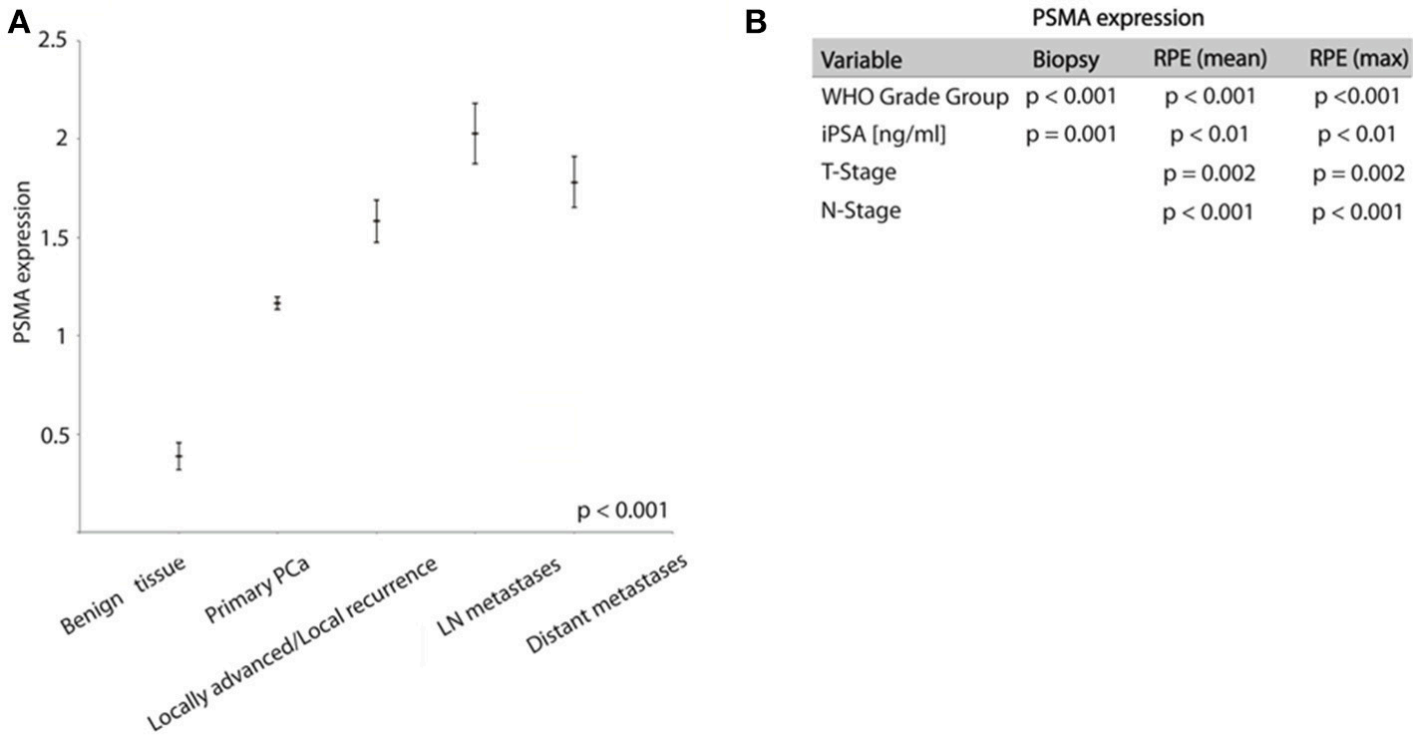
**(A)** Graph showing PSMA expression in different prostate tissue types (mean ± SEM). **(B)** Chi-Square test results analyzing association between PSMA expression and shown variables.

PSMA expression is significantly higher expressed in tumor tissue compared to benign prostatic glands. On biopsies, PSMA expression was detectable in tumor tissue and benign glands in 87 and 16% of cases, respectively. Comparing the PSMA expression during PCa progression, Figure 2A shows increasing PSMA expression in lymph node metastases, locally advanced/recurrent tumors and distant metastases compared to primary tumors, and lowest expression in benign prostatic glands (ANOVA *p* < 0.001, Figure 2A).

High PSMA expression is significantly associated with high grade groups and high iPSA level on both pre-operative biopsy and RPE (Table 2 and Figure 2B). Furthermore, increasing PSMA expression on RPE specimens are associated to higher T-stage and positive nodal status (Figure 2B).

**Table 2:** PSMA staining by grade group on biopsy, chi-square *p* = 0.001.

**PSMA staining on biopsy**	**WHO grade group 1 [*n* (%)]**	**WHO grade group 2 [*n* (%)]**	**WHO grade group 3 [*n* (%)]**	**WHO grade group 4 [*n* (%)]**	**WHO grade group 5 [*n* (%)]**
0 (negative)	19 (18.3)	3 (6.7)	2 (9.5)	1 (3.3)	2 (11.18)
1 (low)	52 (50.0)	19 (42.2)	5 (23.8)	11 (36.7)	3 (17.6)
2 (medium)	28 (26.9)	19 (42.2)	10 (47.6)	9 (30.0)	10 (58.8)
3 (high)	5 (4.8)	4 (8.9)	4 (19.0)	9 (30.0)	2 (11.8)

PSMA expression on biopsy is congruent with PSMA expression on the corresponding RPE specimen (considering mean expression on RPE *p* = 0.006, considering maximum PSMA on RPE *p* = 0.022).

### PSMA Expression on Pre-operative Needle Biopsies Predicts Disease Recurrence Following Surgery

**Figure 3:**
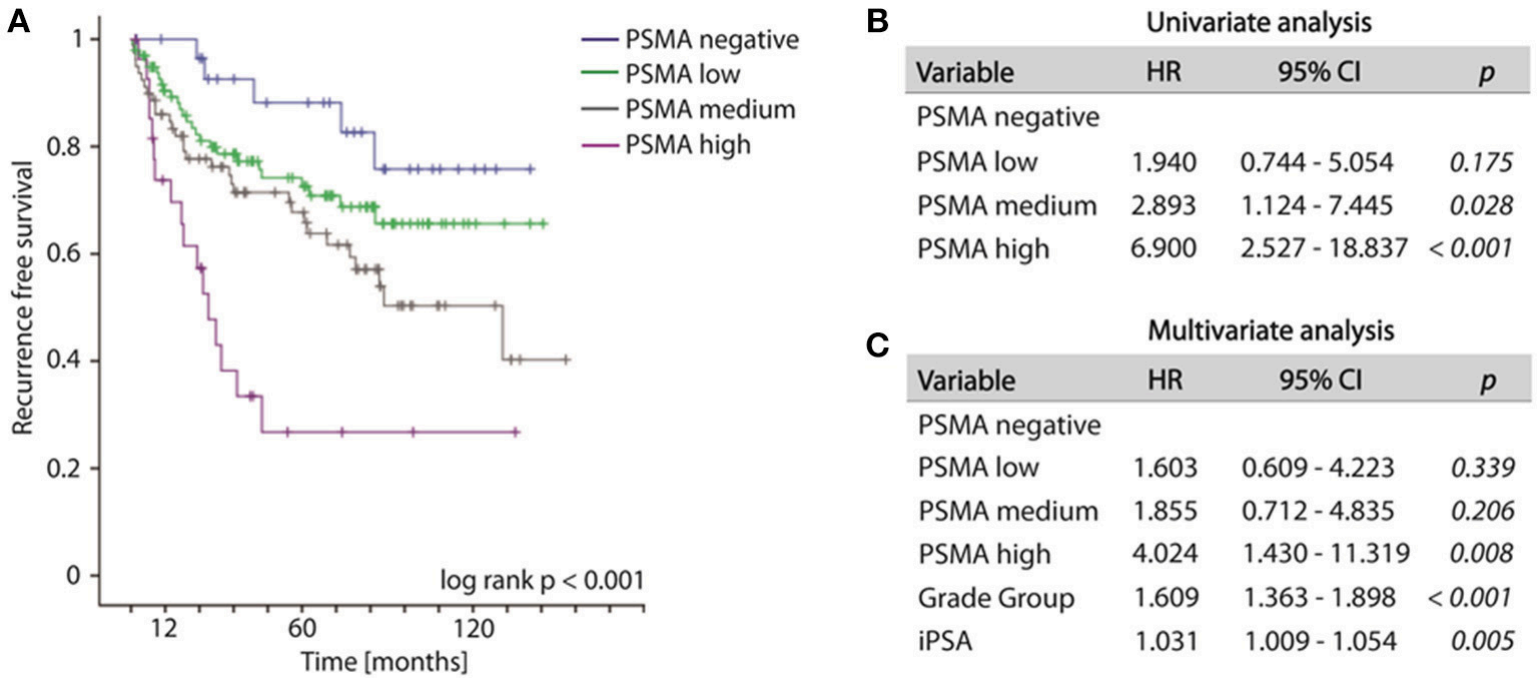
**(A)** Kaplan-Meier curves showing recurrence free survival of patients following radical prostatectomy stratified by PSMA expression on biopsies, log-rank *p* < 0.001. **(B,C)** Cox regression analyses on biopsy cohort for disease recurrence. HR, Hazard ratio; 95%CI,95% confidence interval.

Increased PSMA expression on tumor tissue obtained from biopsy at time point of initial diagnosis is significantly associated with the likelihood of disease recurrence. During the observation period, 79 out of 235 patients (33.6%) developed disease recurrence following RPE. The frequency of disease recurrence was 16.7, 25.7, 39.2, and 60.7% for patients exhibiting no, low, medium or high PSMA expression on pre-operative biopsy, respectively. Kaplan-Meier curve illustrates reduced recurrence free survival with increasing PSMA expression (log-rank test, *p* < 0.001). The 5-year-PSA-recurrence free survival rates are 88.2, 74.2, 67.7, and 26.8% for patients exhibiting no, low, medium or high PSMA expression on pre-operative biopsy, respectively (Figure 3A). Compared to PSMA-negative tumors, low, medium and high PSMA expression is associated with 1.940-, 2.893-, and 6.900-fold incidence of developing disease recurrence following RPE, respectively (univariate Cox-regression, *p* = 0.175, *p* = 0.028, and *p* < 0.001, respectively, Figure 3B). Multivariate Cox-regression adjusting PSMA expression to iPSA blood level at time of diagnosis and grade group on biopsy was used to investigate the potential to predict disease recurrence independently from other prognostic marker. High PSMA expression on biopsy remained significant (*p* = 0.008) in multivariate analysis predicting a 4.024-fold increased risk of disease recurrence in relation to PSMA negative tumors independently from other established prognostic factors (Figure 3C).

### PSMA Expression on Radical Prostatectomy Specimens Predicts Disease Recurrence Following Surgery

**Figure 4:**
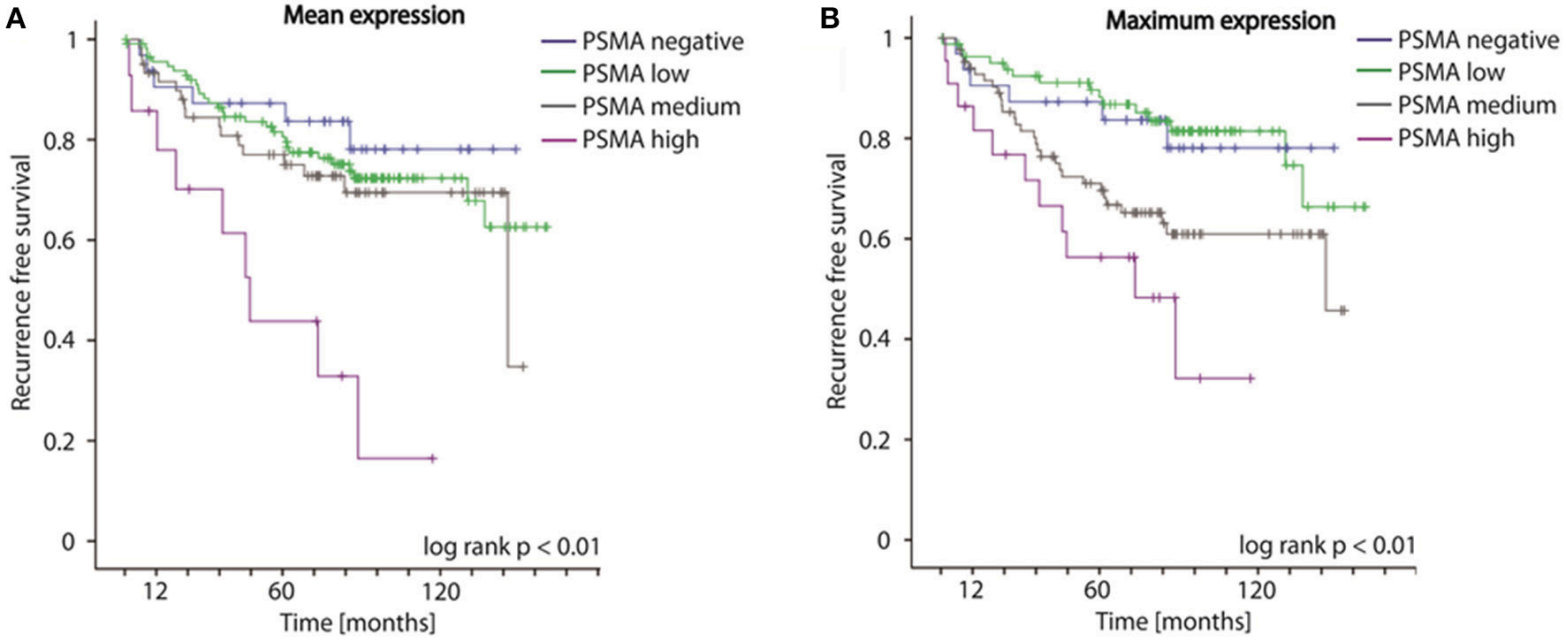
**(A,B)** Kaplan-Meier curves showing recurrence free survival of patients following radical prostatectomy stratified by mean and maximum PSMA expression on RPE specimens, log-rank *p* < 0.01.

Concordantly to our findings on biopsies, both mean as well as maximum PSMA expression considering multifocal tumors of RPE specimens significantly predict disease recurrence following surgery (log-rank *p* < 0.01, Figures 4A,B). The 5-year-disease recurrence free survival rates are 87.3, 81.6, 77.0, and 43.8% for patients exhibiting no, low, medium, or high mean PSMA expression, and 87.3, 89.7, 71.0, and 56.3% for patients exhibiting no, low, medium, or high maximum PSMA expression on RPE (Figures 4A,B). Univariate Cox-regression reveals a 1.267-, 1.950-, and 5.623-fold increased risk in low, medium and high PSMA expressing tumors compared to PSMA-negative tumors (95%CI 0.611–2.626, 0.923–4.122, 2.516–12.565; *p* = 0.525, *p* = 0.08, *p* < 0.001) considering PSMA mean expression, and 0.960-, 2.096-, and 4.102-fold increased risk in low, medium and high PSMA expressing tumors compared to PSMA-negative tumors (95%CI 0.439–2.097, 1.021–4.303, 1.863–9.033; *p* = 0.918, *p* = 0.04, *p* < 0.001) considering maximum PSMA expression. Adjusting PSMA expression to other prognostic factors (grade group, T-, N-, R-status, iPSA blood level), multivariate Cox-regression reveals no significant hazard ratio considering both mean and maximum PSMA expression on RPE.

## Discussion

PCa is characterized by a highly variable disease course, thus stratifying patients into risk groups for individual treatment decisions is still a major clinical challenge. Parameters that are independent from grade group and PSA are urgently needed to improve prognosis at time of initial diagnosis. Considering that PSMA expression on RPE specimens significantly associates with patients' outcome, aim of this study was to assess its prognostic value when assessed on biopsies at time of initial diagnosis, i.e., prior therapy decision. Long term goal of this study is consideration of biopsy PSMA level during decision making for risk-adapted PCa management.

Collectively, our results demonstrate a prognostic value of PSMA expression for disease recurrence following curative surgery at time of initial diagnosis on biopsy. A high PSMA expression on biopsy is associated with a 7-fold increased risk of experiencing disease recurrence following curative therapy compared to negative PSMA expression. In multivariate analyses, high PSMA expression on biopsy predicts disease recurrence independently from established prognostic factors such as Gleason grading or iPSA value. The 5-year-recurrence free survival rates are 88.2, 74.2, 67.7, and 26.8 for patients exhibiting no, low, medium or high PSMA expression on biopsy, respectively. The prognostic potential of PSMA was confirmed on RPE specimens. Furthermore, our study confirms previous study results by other working groups: PSMA expression significantly correlates with the established prognostic parameters grade group and PSA and increases during prostate cancer progression (16–18).

Taken together, PSMA qualifies as an additional prognostic factor determinable on prostate biopsies next to PSA serum levels and grade group. PSMA has the potential to discriminate indolent from aggressive disease and subsequently to stratify patients into risk groups at time of diagnosis. The main advantage is its widespread availability in pathology departments since PSMA is already a well-established and routinely used diagnostic marker for IHC analyses for PCa (4).

There are already a few studies attributing PSMA expression a prognostic value with regard to recurrence free survival. However, PSMA expression in these studies was assessed on RPE specimens, i.e., at time of therapy and thus subsequent to risk stratification of the patient (16, 17). Perner et al. demonstrated that high PSMA expression independently predicts PSA recurrence on RPE samples (HR 1.4, 95%CI 1.1–2.8, *p* = 0.017) (16). Minner et al. showed that tumors with a high PSMA expression had a higher risk of biochemical recurrence (*p* = 0.0483) (17).

PSMA also seems to be a marker of cancer progression. Similar to our results, Perner et al. could show that PSMA expression levels significantly differed between benign prostatic tissue, localized PCa and lymph node metastases (16). Also, Queisser et al. and Wright et al. demonstrated an increased PSMA expression in PCa metastases compared to other PCa tissues (4, 8). Notably, PSMA is also overexpressed in prostatic intraepithelial neoplasia (PIN) compared to benign prostatic tissue (20). Jemaa et al. linked the increased PSMA expression in PCa tissues to an increased angiogenesis by performing CD34 staining (21, 22). Nevertheless, this hypothesis needs further validation.

Limitations of the current study are that the analyses are based on a retrospective data set, clinical T-status for multivariate analysis was not available and disease specific survival as endpoint was not assessable due to low frequency of PCa specific deaths in our cohort. We believe that assessing PSMA expression on prostate biopsies has a great potential to be implemented as an additional biomarker in the clinical management of PCa patients.

In conclusion, our data show that PSMA expression assessed by IHC on prostate biopsy has a great prognostic potential at time of initial diagnosis. PSMA is a routinely used marker for PCa in pathology departments and thus ready to use for including in pathological reports. By performing PSMA expression analyses at time of diagnosis, patient stratification into risk groups can be optimized and individual PCa management improved. Patients with high PSMA expression levels in prostate biopsy have a shorter disease free survival following curative therapy.

## Availability Data and Materials

The datasets generated and/or analyzed during the current study are not publicly available due to data security but are available from the corresponding author on reasonable request.

## Author Contributions

MH, SP, AM, and AO study concept and design; RK, VL, CP, and AO acquisition of data; MH, SP, AM, AO, CK, JR-I, DR, CP, SD, VJ, and FB analysis and interpretation of data; CP, MH, AO, and SP drafting of the manuscript; All co-authors critical revision of the manuscript; VS, MH, and AO statistical analysis; SP, MH, and AO obtaining funding; RK administrative, technical, or material support; SP, AM, and JK supervision.

### Conflict of Interest Statement

The authors declare that the research was conducted in the absence of any commercial or financial relationships that could be construed as a potential conflict of interest.
